# Supplementary material for: Study on Extra Virgin Olive Oil: Quality Evaluation by Anti-Radical Activity, Color Analysis, and Polyphenolic HPLC-DAD Analysis
Source: Foods. 2021 Aug 5;10(8):1808. doi: 10.3390/foods10081808 (PMC8392269; doi:10.3390/foods10081808)
Supplement: Supplementary file 1 [file foods-10-01808-s001.zip › foods-1308767-supplement.pdf]

# Study on extra virgin olive oil: quality evaluation by anti-radical activity, color analysis and polyphenolic HPLC-DAD analysis

Francesco Cairone, Stefania Petralito, Luigi Scipione, Stefania Cesa*

Department of Drug Chemistry and Technology, "La Sapienza" University of Rome, P.le Aldo Moro, 5, 00185 Rome, Italy; francesco.cairone@uniroma1.it, stefania.petralito@uniroma1.it, luigi.scipione@uniroma1.it.

*Correspondence: stefania.cesa@uniroma1.it Tel.:+390649913133

| MILL samples | MILL samples | ITA samples | EUR samples |
|---|---|---|---|
| **A1** | **L10** | **Ar** | **Aa** |
| **B1** | **L11** | **Au** | **Ac** |
| **C1** | **L12** | **Ca** | **Ai** |
| **C2** | **L13** | **Cc** | **Be** |
| **C3** | **M1** | **Ce** | **Cf** |
| **L1** | **P1** | **Cp** | **Cm** |
| **L2** | **P2** | **Ct** | **Co** |
| **L3** | **P3** | **Cv** | **Cr** |
| **L4** | **P4** | **Ds** | **Dc** |
| **L5** | **P5** | **Fb** | **Dn** |
| **L6** | **S1** | **Ma** | **Gi** |
| **L7** | **T1** | **Pa** | **Lr** |
| **L8** | **T2** | **Sa** | **Sp** |
| **L9** | **U1** | | |

**Table S1**. Legend of analysed oil samples.

| | **A1** | **B1** | **C1** | **C2** | **C3** | **L1** | **L2** | **L3** | **L4** | **L5** | **L6** | **L7** | **L8** | **L9** |
|---|---|---|---|---|---|---|---|---|---|---|---|---|---|---|
| **$K_{232}$** | 1.72 | 0.41 | 0.14 | 1.97 | 2.32 | -1.21 | 1.40 | 0.16 | 0.10 | 1.96 | 1.95 | 2.07 | 1.71 | 1.71 |
| **$K_{270}$** | 0.14 | 0.15 | 0.06 | 0.20 | 0.28 | -1.26 | 0.27 | 0.04 | 0.03 | -0.03 | 0.19 | -0.64 | -1.04 | -1.04 |
| **$\Delta K$** | -0.004 | -0.007 | -0.001 | -0.009 | 0.001 | -0.003 | 0.031 | -0.003 | -0.002 | 0.053 | 0.001 | -0.037 | -0.039 | -0.039 |
| **Car*** | 0.018 | 0.017 | 0.033 | 0.028 | 0.017 | 0.048 | 0.042 | 0.021 | 0.038 | 0.015 | 0.021 | 0.036 | 0.038 | 0.016 |
| **Chlor**** | 0.013 | 0.001 | 0.002 | 0.021 | 0.009 | 0.030 | 0.069 | 0.012 | 0.028 | 0.006 | 0.015 | 0.039 | 0.029 | 0.012 |

| | | | | | | | | | | | | | | |
|---|---|---|---|---|---|---|---|---|---|---|---|---|---|---|
| **DPPH*** ** | 89.4 | 29.1 | 37.7 | 122.8 | 34.4 | 154.0 | 72.3 | 71.1 | 96.1 | 57.1 | 237.5 | 83.4 | 136.0 | 41.5 |

| | **L10** | **L11** | **L12** | **L13** | **M1** | **P1** | **P2** | **P3** | **P4** | **P5** | **S1** | **T1** | **T2** | **U1** |
|---|---|---|---|---|---|---|---|---|---|---|---|---|---|---|
| **$K_{232}$** | 1.22 | 3.57 | 1.79 | 1.50 | 2.39 | 0.14 | 2.32 | 2.47 | 3.17 | 3.17 | 0.14 | 0.17 | 2.79 | 1.61 |
| **$K_{270}$** | 0.11 | 0.47 | 0.12 | 0.11 | 0.27 | 0.04 | 0.27 | 0.32 | 0.42 | 0.26 | 0.03 | 0.03 | 0.31 | 0.32 |
| **ΔK** | -0.003 | 0.002 | 0.002 | -0.001 | -0.003 | -0.003 | -0.004 | 0.002 | 0.001 | -0.003 | -0.002 | 0.001 | -0.002 | 0.004 |
| **Car*** | 0.008 | 0.002 | 0.021 | 0.115 | 0.018 | 0.061 | 0.018 | 0.031 | 0.005 | 0.031 | 0.014 | 0.023 | 0.018 | 0.005 |
| **Chlor**** | 0.005 | 0.012 | 0.015 | 0.100 | 0.007 | 0.036 | 0.016 | 0.006 | 0.007 | 0.014 | 0.022 | 0.015 | 0.015 | 0.008 |
| **DPPH***** | 57.0 | 8.3 | 51.4 | 43.2 | 14.7 | 115.4 | 7.4 | 71.4 | 135.0 | 85.5 | 42.7 | 92.8 | 39.8 | 28.3 |

| | **Ar** | **Au** | **Ca** | **Cc** | **Ce** | **Cp** | **Ct** | **Cv** | **Ds** | **Fb** | **Ma** | **Pa** | **Sa** |
|---|---|---|---|---|---|---|---|---|---|---|---|---|---|
| **$K_{232}$** | 2.36 | 2.56 | 1.96 | 0.20 | 1.93 | 0.20 | 0.19 | 0.17 | 1.31 | 1.12 | 2.78 | 2.84 | 2.31 |
| **$K_{270}$** | 0.22 | 0.27 | 0.22 | 0.09 | 0.16 | 0.04 | 0.03 | 0.03 | 0.15 | 0.14 | 0.17 | 0.44 | 0.21 |
| **ΔK** | 0.001 | 0.002 | -0.004 | -0.002 | 0.001 | -0.070 | -0.003 | -0.003 | -0.002 | 0.001 | -0.005 | -0.004 | 0.003 |
| **Car*** | 0.018 | 0.015 | 0.030 | 0.023 | 0.016 | 0.021 | 0.029 | 0.022 | 0.020 | 0.034 | 0.040 | 0.019 | 0.033 |
| **Chlor**** | 0.009 | 0.038 | 0.027 | 0.024 | 0.012 | 0.021 | - | - | - | 0.029 | 0.034 | 0.046 | 0.015 |
| **DPPH***** | 26.0 | 15.3 | 224.9 | 25.0 | 259.7 | 266.1 | 17.4 | 17.7 | 198.2 | 91.7 | 38.2 | 13.4 | 293.2 |

| | **Aa** | **Ac** | **Ai** | **Be** | **Cf** | **Cm** | **Co** | **Cr** | **Dc** | **Dn** | **Gi** | **Lr** | **Sp** |
|---|---|---|---|---|---|---|---|---|---|---|---|---|---|
| **$K_{232}$** | -0.39 | 2.66 | -0.25 | -0.01 | 2.63 | 1.96 | 1.66 | 1.95 | 2.41 | 1.52 | 2.04 | -0.04 | 1.84 |
| **$K_{270}$** | 0.03 | 0.21 | 0.04 | 0.05 | 0.16 | 0.15 | 0.12 | 0.19 | 1.46 | 0.20 | 0.25 | 0.04 | 0.18 |
| **ΔK** | -0.003 | -0.004 | 0.001 | -0.003 | -0.005 | -0.005 | -0.003 | 0.001 | 0.032 | -0.051 | 0.046 | -0.002 | 0.005 |
| **Car*** | 0.013 | 0.017 | 0.032 | 0.024 | 0.029 | 0.021 | 0.037 | 0.019 | 0.022 | 0.026 | 0.026 | 0.016 | 0.022 |
| **Chlor**** | 0.013 | 0.014 | 0.015 | 0.010 | 0.029 | 0.013 | 0.032 | 0.014 | 0.018 | 0.022 | - | - | 0.015 |
| **DPPH***** | 40.0 | 17.9 | 54.6 | 31.6 | 85.5 | 184.3 | 54.4 | 53.5 | 40.4 | 38.7 | 49.5 | 43.1 | 143.0 |

**Table S2.** Spectrophotometric data of all analysed samples.
*Carotenoids detected at 470 nm,**Chlorophylls detected at 670 nm and expressed as absorbance of oils solution (100 mg in 5 mL n-hexane); ***expressed as μg equivalents of gallic acid/g of oil

| | A1 | B1 | C1 | C2 | C3 | L1 | L2 | L3 | L4 | L5 | L6 | L7 | L8 | L9 |
|---|---|---|---|---|---|---|---|---|---|---|---|---|---|---|
| *L** | 47.60 | 54.90 | 45.50 | 45.20 | 56.60 | 42.30 | 36.50 | 51.30 | 44.00 | 48.50 | 49.70 | 41.60 | 38.60 | 43.10 |
| *a** | 0.80 | 0.10 | 2.50 | 2.90 | 0.60 | 2.70 | 0.70 | 3.30 | 3.20 | 0.90 | 4.10 | 4.20 | 1.00 | 1.60 |
| *b** | 18.50 | 46.80 | 33.60 | 30.20 | 50.50 | 29.60 | 13.30 | 44.00 | 33.70 | 20.90 | 41.30 | 27.90 | 17.70 | 30.60 |
| $C^*_{ab}$ | 18.50 | 46.80 | 33.70 | 30.50 | 50.50 | 29.70 | 13.35 | 44.10 | 33.90 | 20.90 | 41.50 | 28.20 | 17.80 | 30.60 |
| $h_{ab}$ | 87.70 | 89.90 | 85.80 | 84.60 | 89.40 | 84.70 | 87.00 | 85.70 | 84.50 | 87.70 | 84.30 | 81.40 | 86.60 | 86.90 |

| | L10 | L11 | L12 | L13 | M1 | P1 | P2 | P3 | P4 | P5 | S1 | T1 | T2 | U1 |
|---|---|---|---|---|---|---|---|---|---|---|---|---|---|---|
| *L** | 55.90 | 49.80 | 51.30 | 53.30 | 52.30 | 43.90 | 50.00 | 53.10 | 56.50 | 51.30 | 43.60 | 47.10 | 51.10 | 39.70 |
| *a** | 0.75 | 0.00 | 1.50 | 1.80 | 3.70 | 1.90 | 4.20 | 5.00 | -1.10 | 4.70 | 4.70 | 3.90 | 3.30 | 2.20 |
| *b** | 48.50 | 6.40 | 39.40 | 47.80 | 46.50 | 30.90 | 42.50 | 48.00 | 44.70 | 45.00 | 32.30 | 35.70 | 43.90 | 24.10 |
| $C^*_{ab}$ | 48.50 | 6.40 | 39.40 | 47.80 | 46.60 | 30.90 | 42.70 | 48.30 | 44.70 | 45.20 | 32.70 | 35.90 | 44.00 | 24.20 |
| $h_{ab}$ | 89.20 | 89.90 | 87.90 | 87.90 | 85.30 | 86.50 | 84.40 | 84.00 | 91.40 | 84.00 | 81.60 | 83.80 | 85.80 | 84.90 |

| | Ar | Au | Ca | Cc | Ce | Cp | Ct | Cv | Ds | Fb | Ma | Pa | Sa |
|---|---|---|---|---|---|---|---|---|---|---|---|---|---|
| *L** | 50.30 | 53.50 | 48.00 | 49.90 | 46.30 | 49.80 | 51.40 | 50.80 | 46.40 | 55.50 | 47.70 | 49.20 | 52.50 |
| *a** | 3.70 | 1.30 | 4.90 | 4.90 | 1.70 | 4.80 | 3.60 | 4.60 | 2.70 | 4.40 | 0.80 | 3.60 | 3.20 |
| *b** | 42.70 | 46.40 | 39.30 | 42.40 | 19.00 | 42.40 | 44.10 | 43.60 | 25.70 | 52.10 | 9.10 | 40.40 | 46.50 |
| $C^*_{ab}$ | 42.80 | 46.40 | 39.60 | 42.60 | 19.10 | 42.60 | 44.30 | 43.90 | 25.90 | 52.20 | 9.20 | 40.60 | 46.60 |
| $h_{ab}$ | 85.00 | 88.40 | 82.90 | 83.40 | 85.10 | 83.60 | 85.30 | 84.00 | 84.00 | 85.10 | 85.10 | 85.20 | 86.30 |

| | Aa | Ac | Ai | Be | Cf | Cm | Co | Cr | Dc | Dn | Gi | Lr | Sp |
|---|---|---|---|---|---|---|---|---|---|---|---|---|---|
| **L*** | 51.80 | 52.20 | 52.10 | 52.80 | 55.70 | 51.40 | 44.00 | 51.40 | 49.30 | 51.40 | 50.60 | 51.70 | 53.30 |
| **a*** | 3.80 | 2.80 | 3.70 | 3.30 | 4.50 | 3.60 | 3.20 | 3.60 | 4.70 | 4.00 | 3.70 | 3.70 | 2.00 |
| **b*** | 45.10 | 45, 6 | 45.20 | 46.50 | 51.90 | 44.10 | 31.00 | 44.10 | 41.50 | 44.70 | 43.30 | 45.00 | 47.40 |
| **C*ab** | 45.30 | 45.70 | 45.30 | 46.60 | 52.10 | 44.30 | 31.20 | 44.30 | 41.70 | 44.80 | 43.50 | 45.20 | 47.40 |
| **hab** | 85.20 | 86.50 | 85.30 | 86.00 | 85.10 | 85.30 | 84.10 | 85.30 | 83.40 | 84.80 | 85.10 | 85.30 | 87.60 |

**Table S3.** Colorimetric data of the analysed samples.

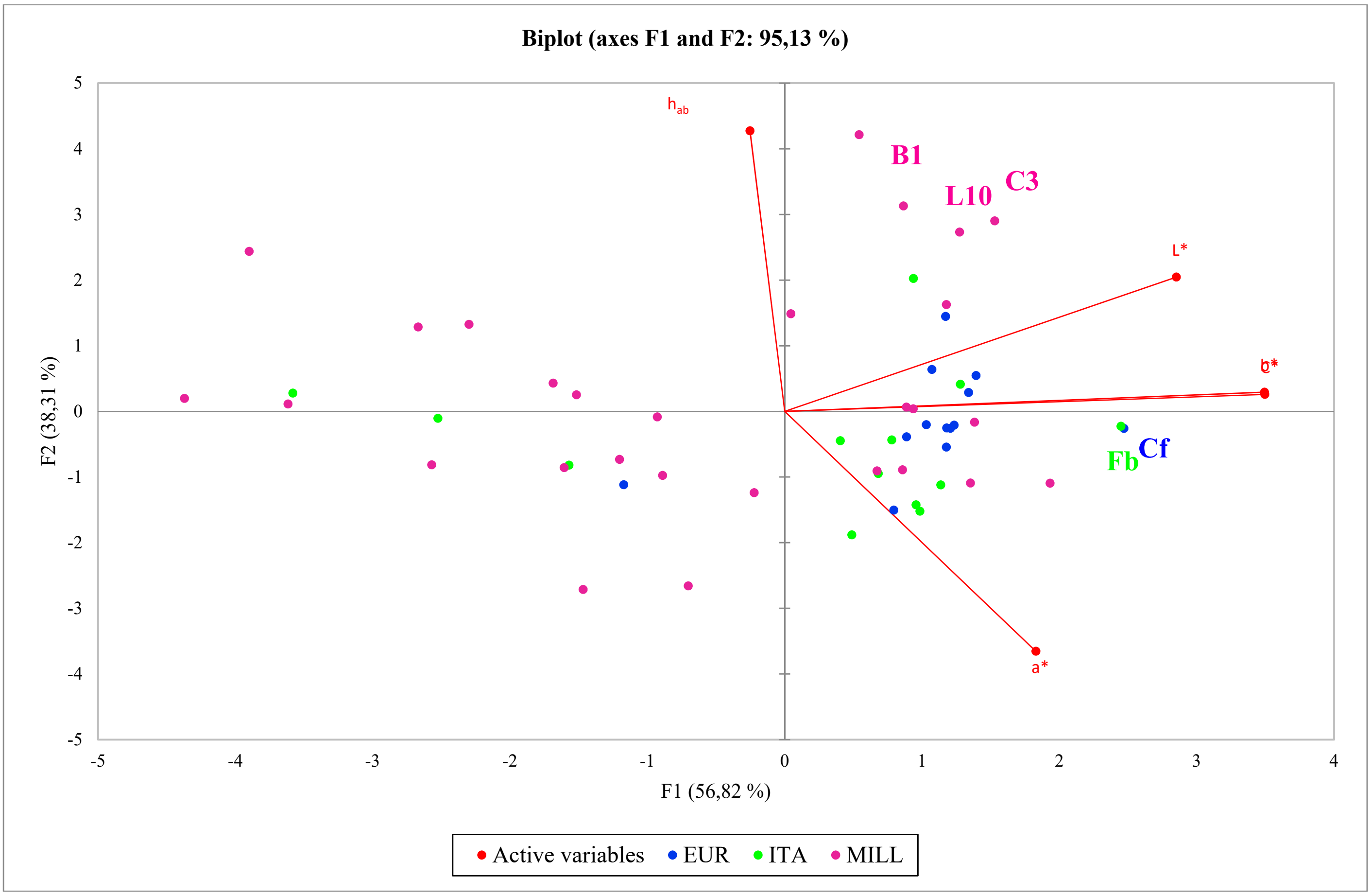


**Figure S1**. Principal component analysis (PCA) of all analysed samples.

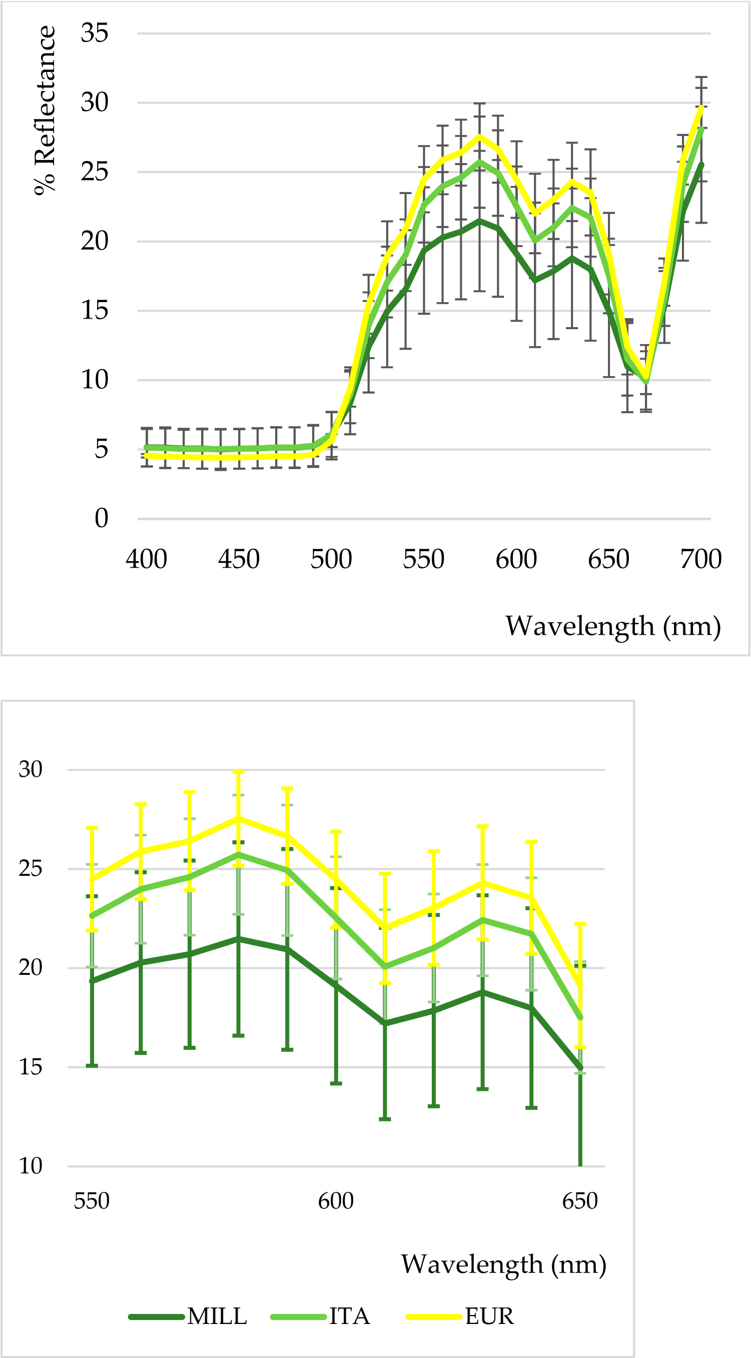


**Figure S2.** Reflectance curves of selected samples.

| | A1 | B1 | C1 | C2 | C3 | L1 | L2 | L3 | L4 | L5 | L6 | L7 | L8 | L9 |
|---|---|---|---|---|---|---|---|---|---|---|---|---|---|---|
| **Hydroxytyrosol** | 32.2 | 4.88 | 3.56 | 35.6 | 1.27 | 3.43 | 5.68 | 6.12 | 5.19 | BDL | 4.98 | 14.4 | 19.7 | 3.8 |
| **Tyrosol** | 39.1 | BDL | 10.5 | 8.84 | 5.76 | 3.84 | 19.7 | 6.53 | 5.78 | 3.31 | 7.08 | 11.9 | 7.91 | 8.89 |
| **Ferulic acid** | BDL | BDL | BDL | BDL | 0.32 | BDL | 0.42 | 0.44 | BDL | BDL | BDL | BDL | BDL | 0.56 |
| **Oleuropein and derivatives** | 30.43 | 22.93 | 4.23 | 5.22 | 22.56 | 16.5 | 9.77 | 18.54 | 13.16 | 8.58 | 3.33 | 6.48 | 53.24 | 3.54 |
| **Flavonols** | 0.99 | 0.63 | BDL | BDL | 0.64 | 0.06 | 0.12 | 0.21 | 0.7 | 0.27 | BDL | BDL | 0.71 | BDL |

| | L10 | L11 | L12 | L13 | M1 | P1 | P2 | P3 | P4 | P5 | S1 | T1 | T2 | U1 |
|---|---|---|---|---|---|---|---|---|---|---|---|---|---|---|
| **Hydroxytyrosol** | 4.64 | BDL | 13.10 | 3.65 | 2.95 | 8.34 | BDL | 5.84 | 3.07 | BDL | 2.28 | 10.70 | 13.50 | BDL |
| **Tyrosol** | 1.75 | 3.66 | 5.16 | 2.32 | 1.45 | 13.80 | 2.66 | 11.50 | 16.60 | 9.24 | 15.30 | 7.07 | 15.90 | 5.23 |
| **Ferulic acid** | BDL | BDL | 4.09 | BDL | BDL | 1.33 | BDL | BDL | 0.71 | BDL | BDL | BDL | BDL | BDL |
| **Oleuropein and derivatives** | BDL | 14.50 | 5.36 | 16.47 | 3.32 | 41.20 | 6.48 | 2.82 | 22.42 | 13.46 | 5.98 | 22.20 | 52.33 | 4.07 |
| **Flavonols** | BDL | 0.18 | 0.03 | 0.24 | 0.12 | 0.57 | 0.04 | 0.09 | 0.26 | 0.24 | 0.23 | BDL | 0.17 | 0.58 |

| | Ar | Au | Ca | Cc | Ce | Cp | Ct | Cv | Ds | Fb | Ma | Pa | Sa |
|---|---|---|---|---|---|---|---|---|---|---|---|---|---|
| **Hydroxytyrosol** | 14.10 | 9.50 | 6.01 | 4.71 | 6.06 | 13.30 | 20.20 | BDL | 15.40 | 1.75 | 16.60 | 2.36 | 12.50 |
| **Tyrosol** | 8.36 | 13.50 | 11.60 | 12.50 | BDL | 2.22 | 27.20 | 11.50 | 22.60 | 2.52 | 12.70 | 7.72 | BDL |
| **Ferulic acid** | 0.86 | 0.77 | 0.91 | 0.89 | BDL | 0.50 | 1.04 | 0.83 | BDL | BDL | 0.36 | 0.46 | 1.04 |
| **Oleuropein and derivatives** | BDL | 24.80 | 37.58 | 30.40 | 23.70 | 56.20 | 55.20 | 16.95 | 70.40 | 0.82 | 44.50 | 18.59 | 43.00 |
| **Flavonols** | 0.25 | 0.41 | 0.50 | 0.18 | 0.44 | BDL | 0.52 | 0.30 | 0.23 | 0.13 | 0.32 | 0.63 | 1.33 |

| | Aa | Ac | Ai | Be | Cf | Cm | Co | Cr | Dc | Dn | Gi | Lr | Sp |
|---|---|---|---|---|---|---|---|---|---|---|---|---|---|
| **Hydroxytyrosol** | 5.38 | 4.68 | 5.58 | 17.10 | 8.80 | BDL | 13.00 | 10.10 | 9.04 | 10.50 | 9.25 | 10.90 | 9.04 |
| **Tyrosol** | 5.60 | 31.40 | 9.03 | 14.60 | 4.78 | 4.87 | 20.00 | 13.80 | 10.50 | 13.20 | 11.60 | 9.49 | 22.70 |
| **Ferulic acid** | 0.58 | BDL | 0.71 | 0.70 | BDL | BDL | BDL | 0.50 | 0.34 | 0.30 | 0.28 | 0.64 | 1.49 |

| | | | | | | | | | | | | | |
|---|---|---|---|---|---|---|---|---|---|---|---|---|---|
| **Oleuropein and derivatives** | 7.54 | 12.29 | 26.00 | 24.10 | 2.17 | 9.13 | 21.70 | 21.42 | 33.20 | 21.30 | 20.22 | 69.60 | 21.24 |
| **Flavonols** | 0.05 | 0.05 | 0.24 | 0.53 | BDL | 0.33 | 0.28 | 0.30 | 0.48 | 0.28 | 0.46 | 1.29 | 0.64 |

**BDL*** below detection limit

**Table S4.** HPLC-DAD analysis of the analysed samples.

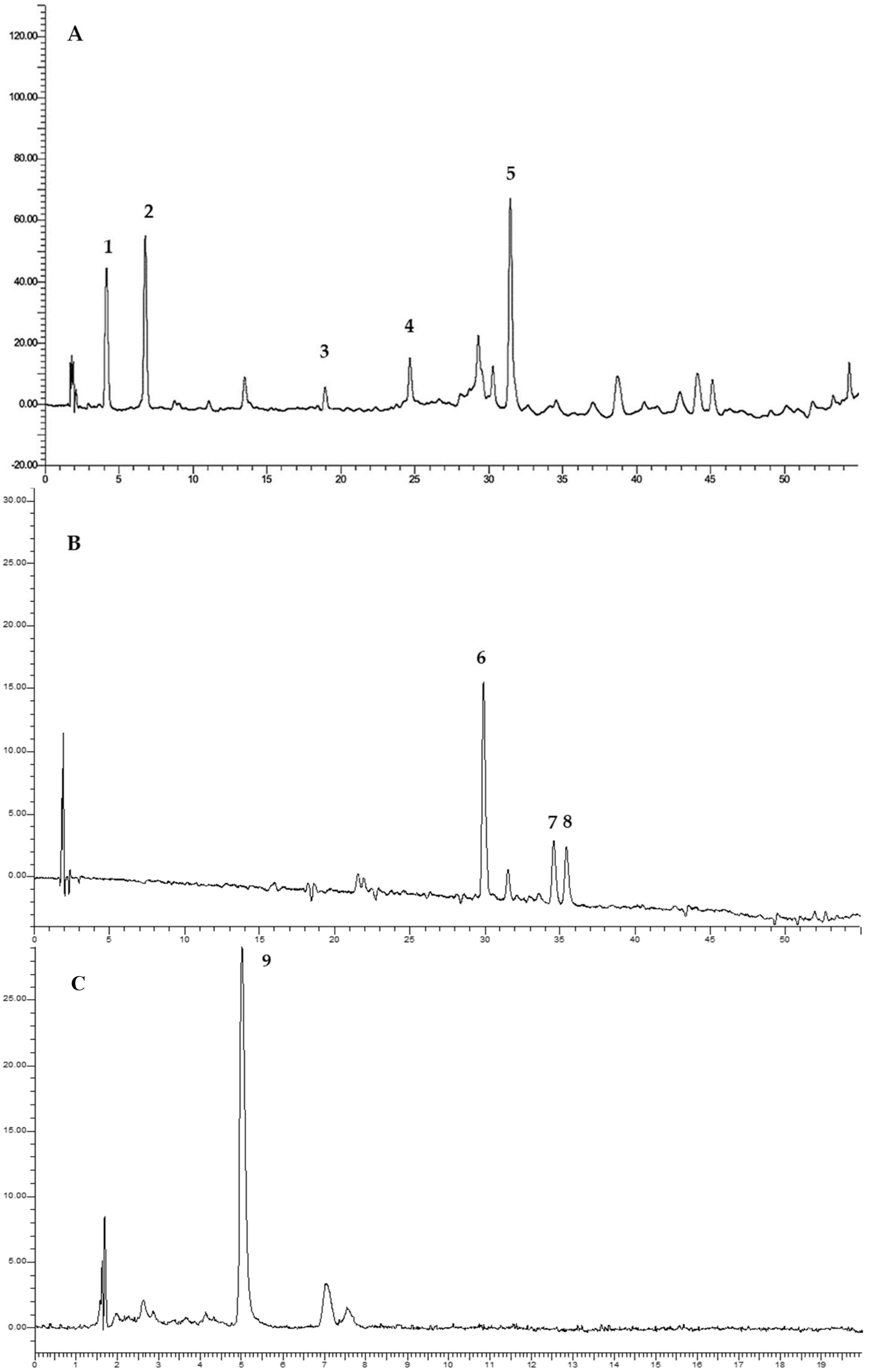


**Figure S3.** Example chromatograms of a selected sample: (Panel A, 280 nm, hydroxytyrosol (1), tyrosol (2), ferulic acid (3), oleuropein (4) and oleuropein derivative (5); Panel B, 360 nm, quercetin-3-D-galactoside (6), quercetin derivative (7), kaempferol derivative (8); Panel C, 450 nm, lutein (9)).

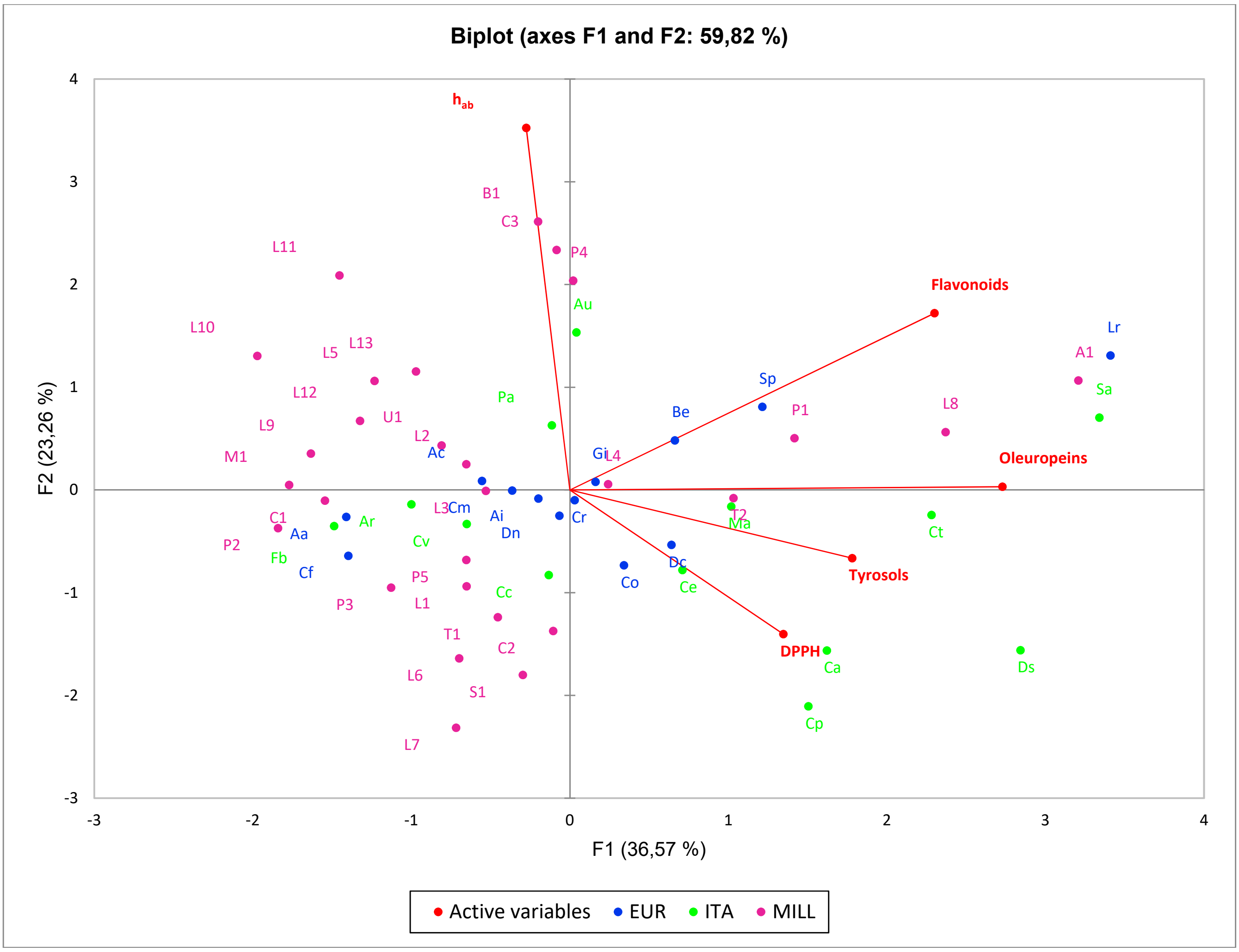


**Figure S4.** Principal component analysis (PCA) correlating quality variables with $h_{ab}$.

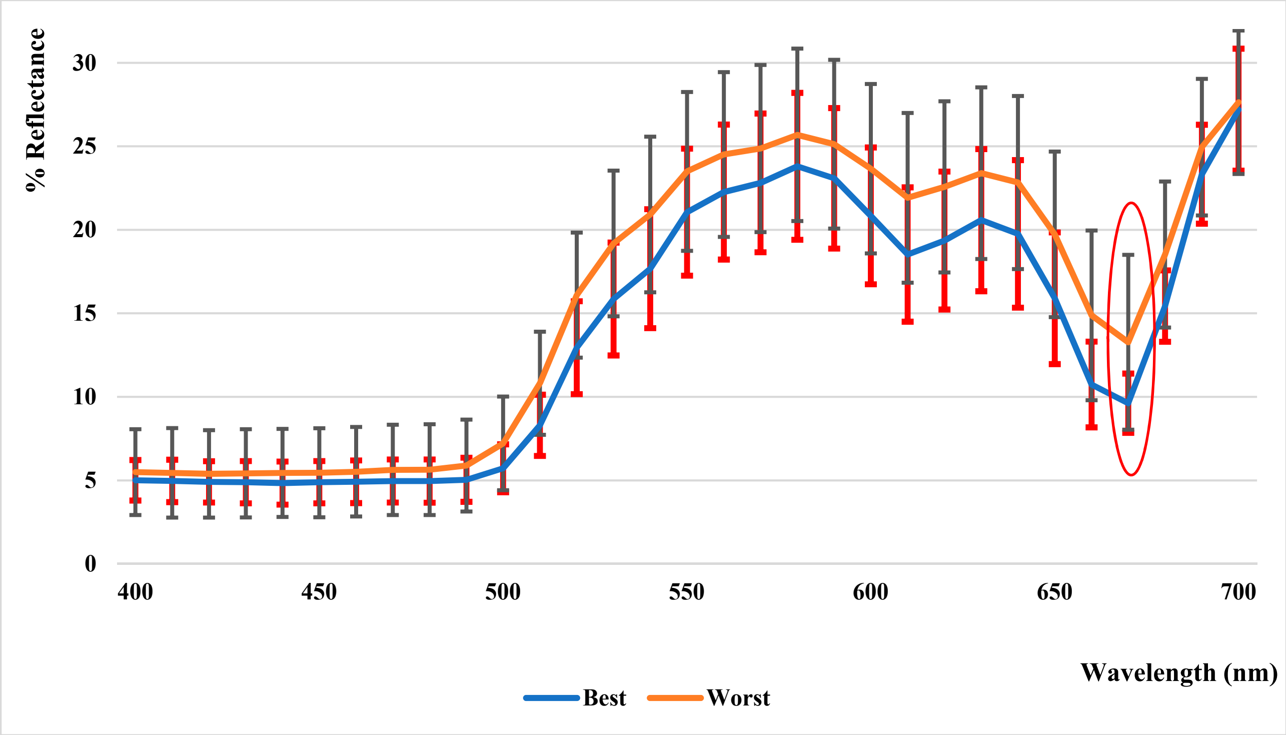


**Figure S5.** Reflectance curves of the two individuated clusters, associated to best or worst oil quality
